# Miners and Minors: The Impact of Mineral Resource Booms on Female Underage Employment

**DOI:** 10.21203/rs.3.rs-8078382/v1

**Published:** 2025-12-01

**Authors:** Sayahnika Basu, Valerie Mueller

**Affiliations:** James Madison University; Arizona State University

**Keywords:** mining, resource curse, human capital, female employment

## Abstract

Resource booms are often associated with adverse distributional effects across economies. We exploit temporal and spatial variation generated by the copper boom in the 2000s to measure the effect of mineral resource extraction on human capital investment in Zambia. Combining data from repeated cross-sections of households and mines, we find that adolescent girls near mines have lower school attendance and higher engagement in paid work. We argue that the main pull factor is the increase in the demand for labor among sectors where women typically dominate. As more adult women marry and married women live in households that benefit from the wealth generated by spouses, adolescent girls fill the gap previously met by adult women in the labor market. Our findings suggest that resource booms induce inequitable distributional effects across generations of women.

## 1 Introduction

Technological consumerism and increasing requirements to reach carbon zero objectives place mineral resource-abundant countries in precarious circumstances (Dayaram, 2022; Fernyhough, 2023). Historically, the internal value of resources to low-income countries was approached with great skepticism, as it was believed declines in long-term growth were attributable to coinciding changes in manufacturing activities (Sachs and Warner, 1995, 1999, 2001). Recent applications of the *resource curse hypothesis* find the welfare implications are not always negative (see Cust and Poelhekke, 2015, for review) and the transmission channels, e.g., adjustments in local sectoral employment (Glaeser et al., 2015; Paredes et al., 2015; Allcott and Keniston, 2018) or infrastructural investments (Michaels, 2011), depend on the national context and resource under evaluation. The social costs of resource extraction are a growing subject of concern as the mining of minerals can affect the environment (Aragón and Rud, 2016), violence (Berman et al., 2017), health (Von der Goltz and Barnwal, 2019), and exacerbate inequality (Marchand and Weber, 2018; Baum and Benshaul-Tolonen, 2021).

We consider the distributional consequences of the 2003 copper boom on human capital investment in Zambia. As a major supplier in Africa (70%) (Kambole and Kambani, 2019), Zambia derives half of its revenue from exporting copper. The few studies in which focus on this isolated boom, country, and resource detected a relative increase in wealth in locales proximate to mines (Wilson, 2012; Zabré et al., 2021; Coulibaly et al., 2024). Although the volume of production creates spillover effects to non-tradeable sectors in Latin America (Aragón and Rud, 2013), the economic benefits of mineral extraction remain at the extensive (rather than intensive) margin in Africa (Mamo et al., 2019). Local wealth effects manifest after five years and engender perverse effects on living standards. This calls for additional research studying the potential drivers of inequality among vulnerable demographic groups (Mamo et al., 2019).

Positive wealth shocks can bear mixed impacts on educational investments. Programs that target poor households align incentives to increase educational investments (De Hoop and Rosati, 2014). Yet, the magnitude of the wealth shock generated from mining activities can pull children into the labor force (Santos, 2018; Zabsonré et al., 2018; Ahlerup et al., 2020; Mejía, 2020). Localized economic growth provides strong incentives for children and adolescents to work off-farm when the income elasticity is zero (Edmonds, 2005; Pellerano et al., 2020) and when the returns to secondary education remain stagnant (Kochar, 2004; De Brauw and Giles, 2017).^1^ Gender differences in schooling outcomes are rarely explored. We posit that girls incur greater losses in human capital as they are the closest substitutes to the adult women who disengage in service-oriented work (Kotsadam and Tolonen, 2016; Benshaul-Tolonen, 2024; Aguilar-Gomez and Benshaul-Tolonen, 2023).

Using the location of mines established prior to the boom and information from two censuses, we employ a difference-in-difference strategy to examine the change in the demographic composition of the workforce amid the copper boom in Zambia. We find declines in adult female labor participation near copper mines parallel to those in the U.S. Gold Boom (Aguilar-Gomez and Benshaul-Tolonen, 2023). We extend the analysis to show, in result, girls within proximity of a mine are less likely to attend school and more likely to engage in paid work. Although the schooling attendance of boys close to mines is also affected, they are no more likely to engage in paid work at the extensive margin. Such effects may occur when the allocation rules for domestic work change as the perceptions of the economic value of girls (relative to boys) improve (Covarrubias et al., 2012; Björkman-Nyqvist, 2013).

Our research contributes to broader themes in environmental and gender economics. First, child labor may increase both with adverse environmental shocks and resource booms, but the incentives underlying the decisions vary. Previous work suggests the former may be driven by increased labor demand to maintain agricultural yields (Beegle et al., 2006), while the latter stems from the relative gains in sectors linked to resource development (Kochar, 2004). In result, positive income shocks can increase child labor force participation (Chong and Yáñez-Pagans, 2019) rather than decrease it (De Hoop and Rosati, 2014). Ultimately, parental preferences in human capital investment, allocation rules across labor activities, and the extent child and adult labor used in specific activities are complements or substitutes influence these decisions which can vary by context (Soares et al., 2012; Dillon, 2013; Pellerano et al., 2020). Understanding how these diverse factors influence parental educational investments is crucial to inform policies aimed at retaining students and increasing enrollment particularly in resource-rich areas.

Second, social norms are likely to interfere with investments during resource booms that particularly benefit women. For example, the rise in economic growth may be insufficient to preserve overall female labor force participation as long as women’s mobility and employment continue to be restricted (Klasen, 2019; Borrowman and Klasen, 2020; Jayachandran, 2021). Moreover, when the growth in female-intensive sectors around resource booms lacks appreciation for literacy, numeracy, and other skills acquired from attending school, drivers of labor demand (in addition to labor supply) reinforce girls’ involvement in the workforce (Heath and Mobarak, 2015). These limitations in occupational mobility ultimately affect women’s agency to make decisions, future employment, and their overall well-being (Luke and Munshi, 2011; Field et al., 2021).

In the next section, we provide some background on copper mining in Zambia and conceptualize through which channels mining impacts human capital investment and labor market dynamics. In Section 3, we present and summarize the various sources of data analyzed, showcasing that temporal trends in the labor market are likely to favor the employment of girls relative to boys given the growth in female-oriented sectors. We then review the empirical strategy and identification in Section 4. Section 5 presents the main results and dispels alternative rationales for the observed effects on girls’ schooling and labor. Section 6 concludes.

## 2 Background and Conceptual Framework

### 2.1 Copper Mining in Zambia

Copper mining forms the cornerstone of the Zambian economy. Around the time of the study, copper and cobalt, a by-product of copper, constituted 75% of Zambia’s export earnings and the value of gross copper exports was greater than 30% of GDP (Lippert, 2014). However, Zambia remained a small player in the international copper market. The country’s share of world copper production was below 4%, and therefore, rendered minimal impact on global market prices (World Bank, 2011).

The mining sector in Zambia has undergone two major transitions in the last century: the mines were nationalized shortly after independence in 1964 and re-privatized in the late 1990s in response to operational inefficiencies, falling per capita GDP, and increased pressure from the international donor community. The majority of Zambia’s copper deposits is located in the Copperbelt, a mineral-rich region in the north that extends into the Katanga region of the Democratic Republic of Congo. The Copperbelt has among the wettest and deepest mines in the world, which require very import- and capital-intensive technologies to operate (Adam and Simpasa, 2010). The unfavorable geology of the Copperbelt combined with the high sunk costs in the mining industry implies that foreign mining companies have strong incentives to react to changes in the price of copper and adjust production accordingly.

Driven by a surge in demand, loose monetary policy, and a weak dollar, the world price of copper rapidly increased in 2003 and 2006 after a decade of reduced volatility. The price shock was exogenous to Zambia and largely unexpected: limited market share implies Zambia is a price taker in the international copper market. In response to the shock, annual copper production in Zambia more than tripled to around 820,000 metric tons in 2010. With rapidly increasing prices and production volumes, gross earnings of copper and cobalt exports exploded between 2003 and 2010, reflecting increased investment in technology, exploration and production capacity (Lippert, 2014). The resource boom was large in absolute terms with windfall estimates ranging between 86% and 110% of 2002 Zambian GDP (Lippert, 2014).

### 2.2 Mining and Human Capital Investment

Resource booms are positively associated with economic growth (Sachs and Warner, 1999; Michaels, 2011), health spending (Acemoglu et al., 2013), and employment (Paredes et al., 2015). However, they also have diverse, distributional impacts across various demographic groups. In particular, the resource curse literature documents the ill effects of resource booms in resource-abundant countries which entrap the poor into poverty (Sachs and Warner, 2001). An additional distributional impact we consider in this paper is on the human capital investment of children.

Resource booms produce disparate incentives for parents to invest in their children’s education. On the one hand, the increase in household wealth can redirect basic consumption towards human and physical capital investments (Glewwe and Jacoby, 2004; Mejía, 2020). On the other hand, these shocks raise the opportunity cost of education among the liquidity-constrained, encouraging adolescents to seek employment rather than education (De Brauw and Giles, 2017). Overarching evidence supports that the latter driver outweighs the former amid resource booms. For example, Mosquera (2022) finds that the oil boom in Ecuador lowered college completion rates, and increased low-skill employment. Ahlerup et al. (2020) shows that adults had lower educational attainment if they lived in a gold mining district in their adolescence. Meanwhile, Santos (2018) detects a decrease in school attendance and increase in child labor in Columbia during the gold rush.

Exposure to a resource boom is likely to engender distinct parental decisions from exposure to other forms of income shocks.^2^ Resource booms increase labor demand in the non-agricultural sector. The expected wage differential potentially entices low-income households to select inactive, young workers to participate in the growing labor market rather than further their education. In what follows, we describe the evidence relating how resource booms affect specific labor markets and pose a rationale for why consequences on the labor supply may differ by the sex and age of the individual in an African context.

### 2.3 Mining and Labor Market Dynamics

A resource boom affects the composition of the labor force along multiple dimensions. The resource boom benefits the local economy in which they operate in the form of an increase in local wages, population, and employment. These outcomes in resource-abundant counties are deemed pro-cyclical with oil and gas: they rise during oil and gas booms and fall during busts in the US (Allcott and Keniston, 2018; Gittings and Roach, 2020). Resource booms also contribute to the growth of other sectors through input-output linkages (Allcott and Keniston, 2018; Michaels, 2011). Allcott and Keniston (2018) finds that manufacturing’s procyclicality is driven by locally-traded sub-sectors and those with upstream or downstream input-output linkages to oil and gas. Michaels (2011) discovers oil-abundant counties in the US increase employment not only in mining but also manufacturing. Overall, the positive income shocks in the local region leads to an increase in the demand for consumables and services. The increase in product demand leads to an expansion of these sectors. This often manifests in an overall increase in labor demand.

The resource boom and bust cycles can create differential effects on the labor market outcomes of men and women. Jayachandran (2021) explains that the relationship between female labor force participation and economic development is U-shaped (Sinha, 1965; Boserup et al., 2013; Durand, 2015). Women drop out of the labor force as a country acquires wealth. This is driven by higher household wealth levels and diminishing value of one additional worker as well as social stigma around women participating in the labor market (Jayachandran, 2015). These general findings imply that the rise of extractive industries will affect the composition of the labor force (Benshaul-Tolonen, 2019). In Africa, women’s labor force participation decreases overall in places benefiting from gold mines, which is in line with the aforementioned U-shaped relationship (Kotsadam and Tolonen, 2016). Conditional on remaining in the labor force, however, women move out of the agricultural sector to the service sector (Kotsadam and Tolonen, 2016). Similarly, Aguilar-Gomez and Benshaul-Tolonen (2023) corroborate observed shifts in gender-specific job opportunities in the historical US. During the gold rush, women, on average, were more likely to be married and engage in housework as mining counties experienced high male to female sex ratios. The gold rush drew men into the mining sector, and those women who worked (rather than married) were employed in the service sector. Overall, the emerging literature on the distributional impacts of extractive industries provide evidence of gender segregation in the labor market. Women in proximity to gold mines are typically less likely to be engaged in the labor force. Conditional on working, women shift from the agriculture to service sector, while mining work is mainly reserved for men (Aragón et al., 2018; Corno and De Walque, 2012; Kotsadam and Tolonen, 2016; Maurer and Potlogea, 2021). This broader shift in the labor force composition triggered by growth in the service sector often leads children in liquidity constrained households to participate in the labor market (Basu et al., 2010). Depending on the growth of certain sectors and preferential treatment of sons (Jayachandran, 2023), young girls engage in paid work.

**Figure 1:**
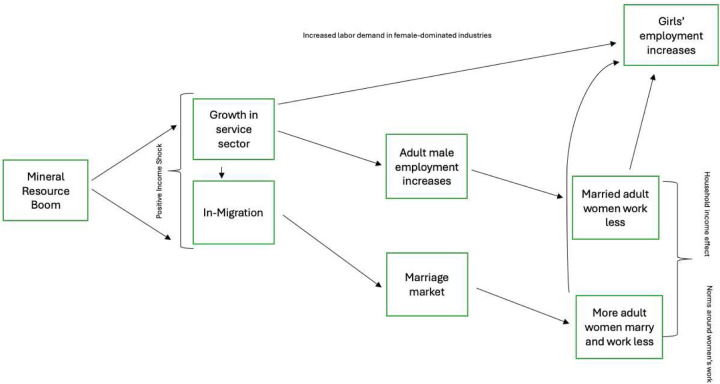
Conceptual Framework

Based on the literature, we develop a conceptual framework in Figure 1 to structure the empirical analysis in the paper. The mineral resource boom is a positive income shock which triggers in-migration and growth in linked sectors through increased demand for consumables. We then examine the following hypotheses. First, girls are less likely to attend school in mining areas and increase their engagement in paid work due to the increased labor demand in sectors that favor women. Second, the boom leads to an increase in migration by marriage-age adults to take advantage of the shock and their labor market participation. Third, women in mining areas are more likely to marry and spouses are more likely to work and bring more income to married women. Together, these two factors contribute to the decline in adult women’s labor force participation near mines due to norms around women’s work and the increased demand for girls’ labor.

## 3 Data and Descriptive Statistics

### 3.1 Zambia Census

Our analysis of individual outcomes is based on a 10% random sample of the 2000 and 2010 censuses of Zambia (Minnesota Population Center, 2020). The sample comprises 996,117 individuals inhabiting 186,225 households in 2000; and 1,321,973 individuals inhabiting 250,805 households in 2010. The constituency of residence for each individual in the censuses can be identified, which is a sub-divisional unit lower than a district.^3^ Each personal record in the census provides information about the individual’s gender, age, educational attainment, occupation and related industry. We further know their relationship to other household members, the size of their household, and other household characteristics reflecting their wealth (i.e., the number of rooms and bedrooms in the household, whether the house obtains a flush toilet or has access to electricity, ownership of various appliances, and digital media connectivity).

Our aim is to evaluate the impact of living within proximate distance of a mine amid a copper boom on adolescent human capital and labor force participation. In addition to restricting our sample to boys and girls (12–18 years old), we limit the analysis to locations within 100 km of the copper mines (in the Copperbelt region of Zambia) in effort to minimize the influence of unobserved heterogeneity and selection bias (Benshaul-Tolonen, 2019; Guimbeau et al., 2023). The main outcomes considered are school attendance and paid work. School attendance is computed based on the survey question which asks students whether they attend school at the time of the survey. Paid work is an indicator that reflects whether the individuals are engaged in self-employment, wage or salary work. The proportion of adolescents attending school rose from 68 to 84 percent over the 10-year period demonstrating an overall positive trend on their educational outcomes (Table 1). Although fewer adolescents engage in paid labor (3 percent in 2000 and 2 percent in 2010), the percentage decline in paid labor over time was greater (33 percent) in magnitude than the percentage increase in schooling attendance (24 percent). Those who shift out of paid work activities may transition into other stages of the life cycle that can interfere with obtaining an education, such as marriage.

To understand how shifts in the composition of the workforce may influence adolescent labor decisions, we further examine how proximity to a mine during the copper boom affects adult labor and marriage market outcomes. According to Table 1, half of the working age sample (between ages 19 to 64) was employed in both years, yet tendencies to become inactive grew from 40 to 48 percent. Mobility, defined as individuals who have been staying in the location for less than or equal to 1 year (short-term migrant) and for 2 to 5 years (medium-term migrant), rose over time. For example, 9 (11) percent of the sample reported moving in the last year (2–5 years) in 2000 compared to 22 (21) percent in 2010.

For the overall sample, the descriptive statistics for the demographic and wealth proxies suggest living standards improved over time. The dependency ratio, which measures the number of household members below the age of 12 and above the age of 64 divided by the number of working age household members, declined and more households obtain a head who has a primary education in 2010 relative to 2000. Moreover, the labor market experienced a substantive change in sectoral composition. While the unemployment rate (the proportion of working age individuals who are unemployed) has been relatively stable at 11 percent, the share of workers employed in manufacturing has risen as well as other unknown industries. Similarly, the share of skilled workers has grown.

### 3.2 Locations of Copper Mines

**Figure 2:**
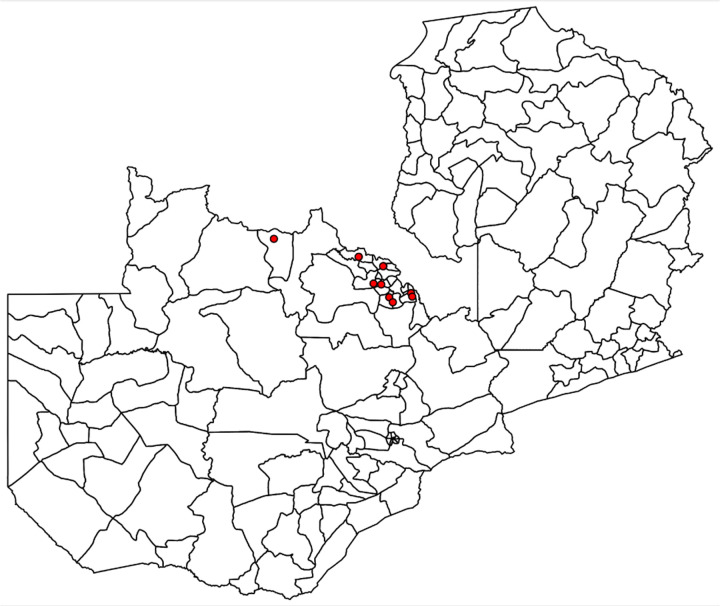
Location of Copper Producing Mines Across Zambian Constituencies Note: The figure shows the location of copper producing mines between the years 1991 and 2002. The mines are primarily located in Copperbelt and North-Western Provinces.

We use a global mining database to extract information on copper mining in Zambia. Mining Intelligence is an online data platform that records information of companies and related mining properties (Costmine Intelligence, 2021). The platform provides the opening and closing dates of each mine, their geographic coordinates, the names and volumes of minerals and metals extracted from the mines, among other information.^4^ Figure 2 displays the location of active copper mines between 1991 and 2002 (prior to the copper boom) in the Copperbelt region. The majority of Zambian copper mines are concentrated in the north-western and copper belt regions until 2002, when mining operations expanded to the central and southern regions where other minerals were also extracted along with copper.

We restrict our focus to the impact of being within close proximity to copper producing mines in the Copperbelt region as it has been the main location of copper mines historically (Sikamo et al., 2016). We exclude the mine in the North-Western province as the distance of the mines to the nearest neighbor constituency is 70km. For each personal census record, we calculate the closest active mine to the individual’s residence using the constituency centroid (the smallest administrative unit provided by the census).^5^ We further reduce the census sample of 437,030 adolescents in 379,384 households to 64,663 adolescents in 67,010 households whom reside within 100 km of copper mine in the Copperbelt region.^6^

### 3.3 Commodity Price Fluctuations and Labor Market Conditions

**Figure 3:**
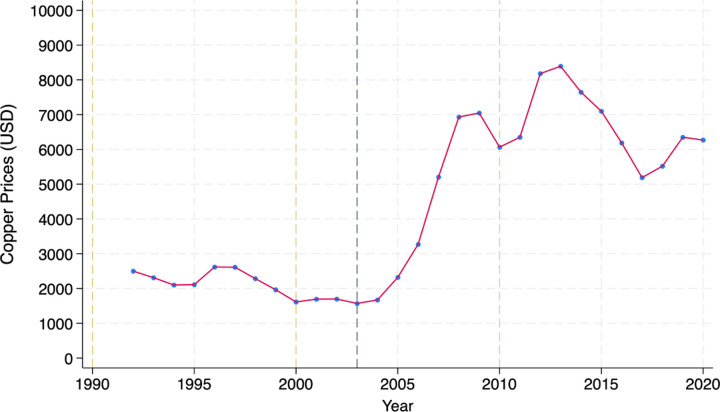
Yearly Trend for International Copper Price Note: The figure shows the two-year moving average of international copper prices, expressed in US dollars between 1992 to 2020.

We use the International Monetary Fund commodity prices database to illustrate the copper price trend over our study period (International Monetary Fund, 2021). The dataset records monthly, quarterly, and annual commodity prices from 1990 to the present date. We use a two-year moving average of international copper prices expressed in US dollars. In Figure 3, we witness a sharp sizable increase post 2003. We define 2003 as the year of the copper boom which lies between the census years of 2000 and 2010.

Next, we describe the labor market conditions over the two census years. In Table 2, we separate the share of employment in each industry by gender to gauge the extent specific labor markets may be gender-segmented. At baseline, the largest share of employment is in agriculture and related industries, followed by wholesale and retail trade and services. There are large differences in the sectoral distribution of employment shares between men and women. In 2000, men were primarily employed in agriculture, services, and mining and extraction, whereas women are employed largely in agriculture, wholesale and retail trade and services.

Employment growth rates across these industries differ for men and women. Men have benefited from activities in construction, wholesale and retail trade, followed by mining and extraction. On the other hand, women have gravitated more towards activities in the following industries: wholesale and retail trade, education, health and social work, and utilities. Taken together, it is possible that the population growth and/or wealth effects inspired by the copper boom may have created an additional demand for services that are typically offered by women laborers in Zambia.

## 4 Empirical Framework

**Figure 4:**
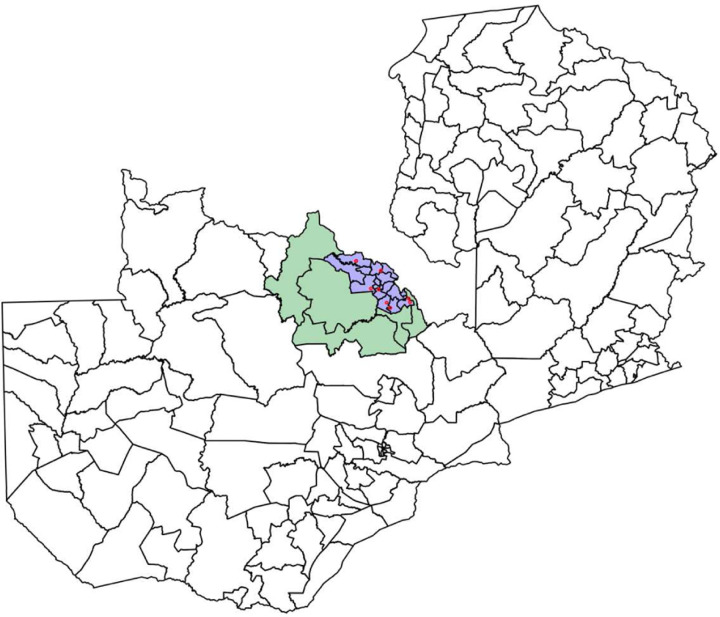
Location of Treatment and Control Constituencies Note: There are 18 constituencies within 30 km of a mine in the Copperbelt region. Five constituencies are within 30–100 km of a mine.

We leverage the global copper price boom to estimate the effect of residing near a mine on human capital investment. Our quasi-experimental approach relies on two sources of temporal and spatial variation: the global copper price boom and household distance to mines. For each household within 100km of a mine, we calculate the distance between the centroid of their residential constituency and the nearest mine. In our preferred specification, the treatment consists of all households located in constituencies within a 30 km buffer of the mine. Figure 4 displays the spatial distribution of the treatment and control constituencies.

Following the literature (Aragón and Rud, 2016; Benshaul-Tolonen, 2024), we use a spatial lag model to identify the distance threshold in which mining activities influence the school attendance of adolescents:

(1) $$y_{idt}=\alpha_{d}+\delta_{t}+\sum_{k=1}^{k=5}\beta_{P}^{k}Post_{idt}\times dist_{idt}^{k}+\beta_{Z}Z_{dt}+\beta_{X}X_{idt}+\epsilon_{idt}$$

The dependent variable, $y_{idt}$, measures whether adolescent i attends school in constituency d in year t. A set of distance binary variables, $dist_{idt}^{k}$, indicate whether the adolescent’s constituency is within the k-distance band from a mine, where values of k (in km) correspond to [0,10], (10, 20], (20, 30], (30, 40] and *>* 40. The variable, $Post_{idt}$ takes value 1 for 2010 census, denoting post-copper boom, zero otherwise. We control for other observed factors that are likely to influence schooling attendance: child and household characteristics in X; labor market conditions Z; as well as region-specific factors, $\alpha_{d}$, and cyclical trends, $\delta_{t}$. The standard errors are clustered at the constituency level.

**Figure 5:**
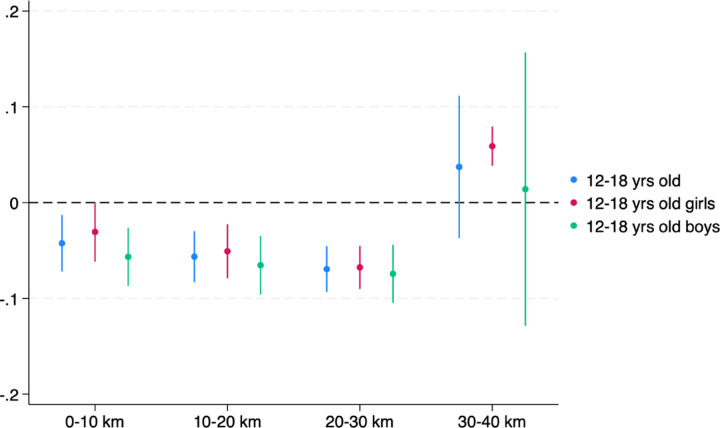
Effect of distance from a mine on school attendance Note: The figure shows the coefficients on the 10-km distance bins and their 95% confidence intervals estimated from the spatial lag model in (1). The omitted category is 40–100km from a mine in the Copperbelt. All constituencies within 100km of a copper mine in the Copperbelt are included in the analysis.

Figure 5 exhibits the estimated coefficients and their 95% confidence intervals for the distance indicators using the pooled and sex-disaggregated adolescent samples. The effect of the copper boom on school attendance is negative and significant up to 30 km of a mine. School attendance declines by 0.1 of a percentage point among boys and girls. The treatment distance is comparable to those identified in previous studies on mining in low- and middle-income countries. For instance, a threshold of: (1) 20 km was applied in a study that quantifies the impact of gold mines on agricultural productivity (Aragón and Rud, 2016); (2) 10–15 km and 15km were used in separate studies that measure the impacts of mining on wealth and health (Von der Goltz and Barnwal, 2019) and female empowerment (Benshaul-Tolonen, 2024); and (3) 5km was used in a study of the effect of mining on women’s agency (Guimbeau et al., 2023).

Table 3 describes the outcomes and features of the adolescent sample at baseline in treatment and control constituencies. Adolescents in the treatment group are positively selected in terms of being more likely to attend school and less likely to engage in paid work. This suggests that slight differences in the composition of the sample might lead us to underestimate the true consequences of the copper boom on human capital investment.

To estimate the impact of mines on school attendance, we refine equation (1) based on the inferences from the spatial lag model analysis:

(2) $$y_{idt}=\alpha_{d}+\delta_{t}+\gamma {Μ}_{d}\times Post_{t}+\beta_{Z}Z_{dt}+\beta_{X}X_{idt}+\epsilon_{idt}$$

where the main difference is we replace the original distance indicators with ${Μ}_{d}$ and $Post_{t}$. The former takes a value 1 if the mine is located within 30 km of the constituency centroid of the household, and the latter indicates whether the survey year is 2010.

## 5 Results

### 5.1 Copper Boom and School Attendance

Table 4 reports the results from specification (2), which is used to estimate the effect of the copper boom on the school attendance of boys and girls ages 12 and 18. Column (1) shows that copper boom led to a 5.3 percentage-point reduction in school attendance among adolescents living within 30 km of a mine. Columns (2) and (3) display the effect of the copper boom on the school attendance of girls and boys, respectively. We find that school attendance has fallen for both. The magnitude of the effect appears higher for girls (7.29%) compared to boys (6.59% ), yet is not significantly different (p-value = 0.977). The coefficients reported in columns (4) - (6) use the same specifications but expand the sample to the entire country as a robustness check. The results are qualitatively similar.

Our findings are consistent with previous work that corroborates a negative impact from a resource boom on human capital investment. For example, the gold boom in Colombia led to reductions in school attendance by 23.9% (Santos, 2018) and lower educational attainment among adolescents (Ahlerup et al., 2020). These results are in contrast with studies that find positive impacts from resource booms. For example, Mejía (2020) detects an increase in primary school enrollment and a reduction in dropout rates at all school levels from the gold boom in Colombia. Agüero et al. (2021) detects improvements in learning outcomes proxied by math and reading scores among students close to mines in Peru. Overall, our finding on school attendance suggests there may be an increase in the opportunity cost of education associated with the copper boom affecting investments in education (De Brauw and Giles, 2017).

To understand the mechanisms underlying the reduction of school attendance among adolescents living near the copper mines, we focus on factors that affect labor markets near mining areas. The reduction in school attendance could be driven by pull factors, such as a higher labor demand in mining or associated industries. The demand for girls’ labor may be in result of adult women working less following the mining boom effects on marriage markets and the labor supply decisions of adult men and women. We explore these mechanisms in the following subsections.

### 5.2 Copper Boom and Labor Market Outcomes

We employ model (2) to estimate the effect of the copper boom on adolescent and adult labor market outcomes. First, we consider the effect of the copper boom on the engagement of adolescents in paid work activities. The results in Table 5 show there is a large marginally significant effect of the copper boom on the girls’ employment. There is no such effect witnessed for boys. Comparing the two coefficients statistically confirms that the effect of the copper boom on the tendency for girls and boys to engage in paid work is indeed statistically significant (p-value = 0.000). The employment results detected for girls emulate those discovered in Santos (2018), where the authors uncover that the gold boom led to an increase in child labor by 9.3% in Colombia.

Next, we examine whether the adult labor market outcomes follow a similar pattern as the adolescents. The dependent variables for the adult labor market regressions are indicator variables for employment and inactivity. Table 6 shows the labor market participation of working age men and women between 19 to 64 years of age. Columns (1) shows a reduction in the employment of working age adults located near the mines after the copper boom. Pooling men and women together in the analysis masks significant heterogeneity in responses. For example, in column (2), we observe that the copper boom actually deterred working age women from engaging in work by 7.4 percentage points while men work 4.3 percentage points more. The last three columns of the table present the effect of the copper boom on inactivity among the pooled sample of working age adults, working age women, and working age men, respectively. We find complementary results for inactivity among the working age population. There is an increase in inactivity among the working age population which is driven by working age women in proximity to the copper mines.

As mentioned in Section 2, the relationship between women’s labor force participation and income follows an inverted-U shape due to norms around women in the workplace. While the copper boom presumably introduced a positive income shock in areas close to the mines, the growth in the demand for work in female-dominated industries might have presumably pulled girls (rather than boys) from liquidity-constrained households into filling the gaps in women’s labor supply. To explore the validity of this channel influencing girls’ schooling decisions, we examine whether the labor responses of women and girls close to the mines varied by the wealth endowment of the household amid the copper boom. We use an indicator for whether the head of household completed a primary education as a proxy for income since the census does not collect such information.

We next compare the heterogeneous effects estimated using the adolescent samples to those using the adult samples. In Table 7, we find an overall reduction in working age employment in households where the head has primary education. This reduction is driven by reductions in the employment of working age women. We do find a positive and marginally significant increase in the employment of working age men. We find complementary results when redirecting the focus to inactivity in the labor market (columns (4) - (6)). The copper boom produces an overall increase in inactivity among working age adults in households where the head has primary education and is close to a mine. This result is again driven by working age women. Columns (2) and (5) suggest that women in wealthier households that are close to the mines are likely to switch from employment to inactivity on the order of 9 percentage points.

Our results show that the composition of the labor force changes in response to the copper boom in markets near mines. Although the employment of men increases amid a copper boom, fewer women (predominantly) from wealthier households will stay in the labor force. We next examine how changes in marital outcomes in result of the copper boom might offer an additional explanation for why girls and adult women adjust their labor supply quite differently.

### 5.3 Copper Boom and Marriage Market

We explore how the copper boom impacted marital decisions for working age adults. To understand adult marriage market dynamics, we focus on the following. We explore whether the copper boom led to an increase in the migration of adults. We focus on the sample that has a higher odds of getting married. This is based on applying the interquartile range of the age of first marriage collected in the 2010 census which suggests that the relevant sample of men and women are those aged 19 to 44. Next, we check whether there is a difference in labor market participation based on the marital status of the unrestricted sample of men and women in treatment areas after the copper boom.

We explore how the copper boom impacted short-term (less than 1 year) and medium-term (2–5 years) migration into mining areas. Previous research illustrates that marital outcomes can increase in mines following a boom due to the influx of wealthier men (Baum and Benshaul-Tolonen, 2021). In columns (1) and (2) of Table 8, we observe a large increase in the short-term migration of men (50.00% relative to the mean) and women (40.28% relative to the mean). However, we do not find any significant changes in medium-term migration. This provides suggestive evidence that an increase in the supply of grooms may influence the marital prospects of adult women and their respective dropout rates from the labor force after the copper boom.^7^

Furthermore, we investigate whether the labor responses of men and women to the copper boom differ by marital status. In column (1) and (2) of Table 9, we show that married women are even less likely to be employed (−0.056 compared to −0.081) and more likely to be inactive (0.047 compared to 0.072) when the copper boom hits. We repeat the analysis using the working age men sample in columns (3) and (4) and the effects are opposite. Married, working age men are more likely to be employed and less likely to be inactive in response to the copper boom.

Overall, we find that the copper boom in Zambia led to a reduction in human capital investment indicated by declines in the school attendance of adolescents. Additionally, there is substitution of labor market participation across working age women and younger adolescent girls due to the increase in labor demand in female-dominated industries, such as the service sector. We also provide suggestive evidence that women leave the labor force when liquidity constraints at the household level are relaxed and when married due to norms around the allocation of their labor outside of the home. Taken together, the results suggest that the copper boom might increase gender inequality in two forms: (1) the effect on wealth and marital outcomes removes women from the labor force due to societal norms; and (2) the increased growth in employment in specific sectors can exacerbate girls’ long-term employment prospects by incentivizing households to take girls out of school and place them prematurely in the labor force.

## 6 Conclusion

This paper contributes to the resource extraction literature by exploring how the copper boom in the 2000s affected human capital investment in Zambia and how changes in labor and marriage markets might have influenced its outcome. We combine repeated cross-sectional household data with mining data to estimate the effect of the copper boom on school attendance and child labor. Girls living near the mines increased their engagement in paid work and withdrew themselves from school post copper boom. We additionally explored the channels in which the resource boom might have created a wedge between the supply and demand for service-oriented jobs, luring households to encourage their adolescent girls to work rather than attend school.

The demand for service-oriented labor came from sectoral growth as well as changes in the dynamics of labor supply induced by the resource boom. Women who would have otherwise been employed in service-oriented jobs were more likely to marry and spouses were more likely to work and bring more income to married women. While adult men increased their employment when living near the copper mines, adult women retracted from the labor market. These effects were stronger for individuals in wealthier households corroborating global evidence which suggests women’s labor force participation suffers in less poor countries due to norms around women’s work (Jayachandran, 2015). Overall, the decline in the adult women’s labor force participation near mines contributed to the excess demand for labor in sectors targeting women, thereby affecting the economic activities of their closest substitutes, adolescent girls.

Our paper highlights a potential, negative externality of a mineral resource boom. The reduction in educational investments (as well as on-the-job training) can jeopardize individual income in the long term and economic development (Schultz, 1961; Becker, 1962; Weisbrod, 1962; Becker et al., 1990). In the present context of the mineral-rich Zambian economy, it is concerning to find lower human capital investment due to rising copper prices. There is an apparent trade-off being made between the income gains in the short term and gains in economic development and growth in the long term. Traditionally, copper and cobalt have been the backbone of Zambia’s economy, constituting over 70% of its export earnings. The Zambian government has ambitious plans to increase copper production to 3 million metric tonnes by 2032, from 760,000 tonnes in 2022.^8^ Future analysis is required to understand the distributional implications of increased copper production at the aggregate and local levels. With its high demographic dividend, the pace of economic development in sub-Saharan Africa will ultimately be determined by young adults. Investments targeting girls will be crucial to combat norms around marriage and labor force participation, secure the future labor force, and promote women’s social and economic empowerment in resource-rich countries.

## Figures and Tables

**Table 1:** Summary Statistics

	*2000*			*2010*		
	Mean	Std. Dev.	Obs.	Mean	Std. Dev.	Obs.
*Adolescent Outcomes*						
School Attend	0.68	0.47	28762	0.84	0.37	35901
Paid Work	0.03	0.17	25791	0.02	0.14	33540
*Working Age Outcomes*						
Employed (Working Age)	0.50	0.50	73393	0.52	0.50	88570
Inactivity (Working Age)	0.40	0.49	73393	0.36	0.48	88570
Short Term Migrant (0–1 years)	0.09	0.29	73393	0.22	0.41	93296
Medium Term Migrant (2–5 years)	0.11	0.31	73393	0.21	0.41	93296
*Demographic and Location Characteristics*						
Married	0.30	0.46	164719	0.28	0.45	202583
Dependency Ratio	112.82	93.87	163669	100.52	82.47	200771
Primary Education (Head)	0.72	0.45	164709	0.81	0.39	202583
Unemployment Rate (Constituency)	10.72	4.64	164719	10.51	3.80	202583
Agriculture (Constituency)	49.95	29.15	164719	46.83	26.22	202583
Manufacturing (Constituency)	14.56	9.98	164719	17.04	9.22	202583
Services (Constituency)	35.49	20.05	164719	35.79	17.26	202583
Other Unknown Industry (Constituency)	0.00	0.00	164719	0.35	0.39	202583
Skilled 19–64 yrs (Constituency)	14.49	6.70	164719	15.34	6.66	202583
Unskilled 19–64 yrs (Constituency)	84.68	6.97	164719	81.36	6.99	202583
Unknown skill 19–64 yrs (Constituency)	0.83	0.38	164719	3.30	0.83	202583

Note: The table shows the summary statistics for the study sample which is restricted within 100km of a copper mine in the Copperbelt region. All outcomes and variables are representative of individuals unless otherwise indicated. The adolescent sample comprises boys and girls that are 12–18 years old, while the work age sample comprises men and women that are 19 to 64 years old. Parenthetical comments (Head) and (Constituency) reflect an attribute of the individual’s head of household or constituency.

**Table 2:** Labor Market in the Copperbelt Region, Zambia

Industry	Employment Share, 2000			Growth Rate (2010 & 2000)		
	Pooled	Men	Women	Pooled	Men	Women
Agriculture, Forest and Fishing	43.05	36.90	54.71	25.33	19.50	32.78
Mining and Extraction	9.74	14.13	1.43	61.29	61.12	64.41
Manufacturing	6.69	8.30	3.62	31.77	31.18	34.30
Electricity and Gases	1.05	1.45	0.29	44.38	38.53	100.00
Construction	2.22	3.24	0.28	214.14	220.06	84.57
Wholesale and Retail Trade	12.32	9.94	16.83	110.77	74.41	151.32
Services	18.59	21.39	13.30	37.02	35.61	41.31
Education	4.27	3.19	6.23	67.89	27.74	106.37
Health and Social Work	2.07	1.46	3.23	73.14	39.87	101.62

Note: Columns (1)-(3) shows the employment shares across different industries for the entire population, men and women respectively for our study sample. Columns (4)-(6) shows the growth rate in employment across different sectors for the entire population, men and women, respectively.

**Table 3:** Baseline Characteristics Across Treatment and Control

	*Treatment*			*Control*		
	Mean	Std. Dev.	Obs.	Mean	Std. Dev.	Obs.
***Child***						
School Attend	0.71	0.45	24491	0.53	0.50	4271
School Attend (Girls)	0.67	0.47	12776	0.47	0.50	2149
School Attend (Boys)	0.75	0.43	11715	0.58	0.49	2122
Paid Work	0.02	0.15	22765	0.09	0.29	3026
Paid Work (Girls)	0.02	0.13	11916	0.10	0.31	1509
Paid Work (Boys)	0.03	0.16	10849	0.08	0.27	1517
***Household***						
Dependency Ratio	108.67	86.70	137296	134.42	122.47	26373
Primary Education (Head)	0.77	0.42	137955	0.49	0.50	26754
***Constituency***						
Unemployment Rate (Constituency)	12.35	3.04	137965	2.30	0.48	26754
Agriculture (Constituency)	41.38	23.70	137965	94.16	1.35	26754
Manufacturing (Constituency)	17.14	8.82	137965	1.23	0.66	26754
Services (Constituency)	41.48	16.09	137965	4.61	1.05	26754
Other Unknown Industry (Constituency)	0.00	0.00	137965	0.00	0.00	26754
Skilled 19–64 yrs (Constituency)	16.78	4.59	137965	2.67	0.66	26754
Unskilled 19–64 yrs (Constituency)	82.28	4.74	137965	97.06	0.71	26754
Unknown skill 19–64 yrs (Constituency)	0.94	0.30	137965	0.27	0.18	26754

Note: The table shows the baseline charactertistics for children, household and constituency level variables for the year 2000 across treatment (0–30 km of a mine) and control (30–100 km of a mine) in Copperbelt.

**Table 4:** Effect of Copper Boom on Children’s School Attendance

	(1) Pooled	(2) Girl	(3) Boy	(4) Pooled	(5) Girl	(6) Boy
Post X Treatment	−0.053^**^ (0.022)	−0.054^**^ (0.023)	−0.053^*^ (0.028)	−0.033^**^ (0.013)	−0.032^**^ (0.014)	−0.032^**^ (0.013)

Difference between girl and boy (p-value)			0.977			0.974

Constituency FE	Yes	Yes	Yes	Yes	Yes	Yes
Observations	63863	32974	30889	122024	62490	59534
Mean Dep. Variable	0.771	0.741	0.804	0.733	0.697	0.771
SD Dep. Variable	0.420	0.438	0.397	0.442	0.460	0.420

The treatment group is 30km and the control group is 30–100km of a copper mine for columns (1)-(3) and the entire country for columns (4)-(6). The sample is for years 2000 and 2010. The dependent variable is the indicator for school attendance. There are individual-level, household-level and constituency-level labor market controls included in the above regressions. The individual-level variables include age and gender of the child. Household-level variables include the dependency ratio and head’s education level. Constituency-level labor market variables include unemployment rate, share of employment in different sectors and skill level of the workers.

* p < 0.1

** p < 0.05

*** p < 0.01.

**Table 5:** Effect of Copper Boom on Children’s Labor Market Participation

	(1) Pooled	(2) Girl	(3) Boy
Post X Treatment	0.010 (0.014)	0.024^*^ (0.014)	−0.004 (0.015)

Difference between girl and boy (p-value)			0.000

Constituency FE	Yes	Yes	Yes
Observations	58644	30357	28287
Mean of Dep. Variable	0.024	0.021	0.027

The treatment group is 30km and the control group is 30–100km of a copper mine between 1990 and 2000. The sample is for years 2000 and 2010. The dependent variable is the indicator for paid work. There are individual-level, household-level and constituency-level labor market controls included in the above regressions. The individual-level variables include age and gender of the child. Household-level variables include the dependency ratio and head’s education level. Constituency-level labor market variables include unemployment rate, share of employment in different sectors and skill level of the workers.

* p < 0.1

** p < 0.05

*** p < 0.01.

**Table 6:** Effect of Copper Boom on Adult Labor Market Participation

	(1) Employed Working Age	(2) Employed Working Age Female	(3) Employed Working Age Male	(4) Inactivity Working Age	(5) Inactivity Working Age Female	(6) Inactivity Working Age Male
Post X Treatment	−0.018^**^	−0.074^***^	0.043^***^	0.017^**^	0.065^***^	−0.037^***^
	(0.008)	(0.011)	(0.014)	(0.007)	(0.013)	(0.011)

Constituency FE	Yes	Yes	Yes	Yes	Yes	Yes
Observations	161953	80069	81884	161953	80069	81884
Mean of Dep. Variable	0.512	0.365	0.656	0.377	0.559	0.199

The treatment group is 30km and the control group is 30–100km of a copper mine between 1990 and 2000. The sample is for years 2000 and 2010. The dependent variable is the indicator for employment and inactivity. There are individual-level, household-level and constituency-level labor market controls included in the above regressions. The individual-level variables include age and gender of the child. Household-level variables include the dependency ratio and head’s education level. Constituency-level labor market variables include unemployment rate, share of employment in different sectors and skill level of the workers.

* p < 0.1

** p < 0.05

*** p < 0.01.

**Table 7:** Effect of Copper Boom on Adult’s Labor Market Participation, Heterogeneity by Head’s Education Level

	(1) Employed Working Age	(2) Employed Working Age Female	(3) Employed Working Age Male	(4) Inactivity Working Age	(5) Inactivity Working Age Female	(6) Inactivity Working Age Male
Post X Treatment	−0.011 (0.009)	−0.040^***^ (0.013)	0.028^*^ (0.016)	−0.013 (0.010)	0.012 (0.016)	−0.050^***^ (0.015)
Primary Educated Head X Post	0.012 (0.011)	0.015 (0.011)	0.027^***^ (0.010)	−0.023^*^ (0.011)	−0.026^***^ (0.007)	−0.043^***^ (0.014)
Primary Educated Head X Treatment	0.036^**^ (0.014)	0.016 (0.019)	0.049^***^ (0.016)	−0.026^**^ (0.012)	−0.018 (0.018)	−0.022^*^ (0.012)
Primary Educated Head X Post X Treatment	−0.017 (0.014)	−0.048^***^ (0.016)	0.004 (0.016)	0.045^***^ (0.013)	0.073^***^ (0.017)	0.029^*^ (0.016)
Primary Education (Head)	−0.012^**^ (0.006)	−0.034^**^ (0.013)	0.006 (0.009)	0.024^***^ (0.007)	0.049^***^ (0.012)	−0.004 (0.007)

Post X Treatment + Primary Educated Head X Post X Treatment	−0.028^**^ (0.012)	−0.088^***^ (0.014)	0.031^*^ (0.017)	0.032^***^ (0.009)	0.085^***^ (0.015)	−0.021 (0.013)

Constituency FE	Yes	Yes	Yes	Yes	Yes	Yes
Observations	161953	80069	81884	161953	80069	81884
Mean of Dep. Variable	0.512	0.365	0.656	0.377	0.559	0.199

The treatment group is 30km and the control group is 30–100km of a copper mine for columns (1)-(3) and the entire country for columns (4)-(6). The sample is for years 2000 and 2010. The dependent variable is the indicator for school attendance. There are individual-level, household-level and constituency-level labor market controls included in the above regressions. The individual-level variables include age and gender of the child. Household-level variables include the dependency ratio and head’s education level. Constituency-level labor market variables include unemployment rate, share of employment in different sectors and skill level of the workers.

* p < 0.1,

** p < 0.05,

*** p < 0.01.

**Table 8:** Effect of Copper Boom on Short-term and Medium-term Migration

	(1) Short-term Male	(2) Short-term Female	(3) Medium-term Male	(4) Medium-term Female
Post X Treatment	0.037^***^ (0.011)	0.029^***^ (0.009)	0.003 (0.009)	−0.001 (0.010)

Constituency FE	Yes	Yes	Yes	Yes
Observations	162260	164427	162260	164427
Mean of Dep. Variable	0.074	0.072	0.075	0.073

The treatment group is 30km and the control group is 30–100km of a copper mine between 1990 and 2000. The sample includes adults between ages 19 to 44 for years 2000 and 2010. The dependent variable is the indicator for short-term (less than one year) and medium term (2 to 5 years) migration. There are individual-level, household-level and constituency-level labor market controls included in the above regressions. The individual-level variables include age and gender of the child. Household-level variables include the dependency ratio and head’s education level. Constituency-level labor market variables include unemployment rate, share of employment in different sectors and skill level of the workers.

* p < 0.1

** p < 0.05

*** p < 0.01.

**Table 9:** Effect of Copper Boom on Adult’s Labor Market Participation, Heterogeneity by Marital Status

	(1) Employed Working Age Female	(2) Inactivity Working Age Female	(3) Employed Working Age Male	(4) Inactivity Working Age Male
Post X Treatment	−0.056*** (0.018)	0.047** (0.019)	0.052** (0.025)	−0.057*** (0.019)
Married X Post X Treatment	−0.025 (0.018)	0.025 (0.022)	−0.004 (0.029)	0.020 (0.021)
Married X Post	0.051*** (0.013)	−0.055*** (0.014)	0.070*** (0.021)	−0.078*** (0.016)
Married X Treatment	−0.028 (0.026)	0.132*** (0.031)	0.153*** (0.021)	−0.062*** (0.014)
Married	−0.099*** (0.023)	0.123*** (0.026)	0.099*** (0.010)	−0.079*** (0.008)

Post X Treatment + Married X Post X Treatment	−0.081*** (0.011)	0.072*** (0.0146)	0.048*** (0.016)	−0.037*** (0.012)

Constituency FE	Yes	Yes	Yes	Yes
Observations	80069	80069	81884	81884
Mean of Dep. Variable	0.365	0.559	0.656	0.199

The treatment group is 30km and the control group is 30–100km of a copper mine between 1990 and 2000. The sample is for years 2000 and 2010. The dependent variable is the indicator for employment and inactivity. There are individual-level, household-level and constituency-level labor market controls included in the above regressions. The individual-level variables include age and gender of the child. Household-level variables include the dependency ratio and head’s education level. Constituency-level labor market variables include unemployment rate, share of employment in different sectors and skill level of the workers.

* p < 0.1

** p < 0.05

*** p < 0.01.

## Footnotes

1 Few governments have addressed this issue through policy response. Peru offers an exception where efforts to redistribute royalties to local governments improved infrastructure and the quality of human capital (Agüero et al., 2021).

2 Earlier work in the child labor literature identified the tendency for parents to withdraw their children from school to work on the farm during a negative production shock in Africa (Cogneau and Jedwab, 2012; Dillon, 2013). Cash transfer programs have benefited children by reducing variability in educational investments in low-income households (De Hoop and Rosati, 2014).

3 An additional census is available in Zambia for 1990. We exclude it from the analysis as it does not provide geographical information at an administrative unit lower than the district.

4 The data on volume of production was unavailable for Zambia and hence we use the production years only.

5 There were 150 constituencies in Zambia at the time of the censuses, and the boundaries remain constant over the study period.

6 Appendix table A1 shows how the characteristics vary across households in the study sample and households in the rest of the country.

7 Unfortunately, we do not have the age of first marriage for individuals in both censuses to explore the impact of the copper boom on marital outcomes. Using the age of first marriage information in 2010, we qualitatively show that individuals closer to mines are more likely to marry post copper boom. In Appendix section table A2, the results indicate that individuals between ages 19 and 44 years are more likely to get married if they are living between 30 km of a mine compared to if they are living between 30 to 100 km of a mine. Additionally, heterogeneity analysis shows that this effect is stronger for individuals from educated households and women.

8 Link to the development plan: Eight National Development Plan 2022–2025

## Appendix

**Table A1:** Summary Statistics: In Sample and Rest of Zambia

	*In Sample*			*Out of Sample*		
	Mean	Std. Dev.	Obs.	Mean	Std. Dev.	Obs.
School Attend	0.77	0.42	64663	0.69	0.46	59397
Paid Work	0.03	0.16	59331	0.05	0.22	49991
Employed (Working Age)	0.51	0.50	161963	0.61	0.49	138675
Inactivity (Working Age)	0.38	0.48	161963	0.34	0.48	138675
Short Term Migrant (0–1 years)	0.16	0.37	166689	0.14	0.34	145220
Medium Term Migrant (2–5 years)	0.17	0.37	166689	0.18	0.38	145220
Married	0.29	0.45	367302	0.29	0.45	357942
Marriage After Boom	0.06	0.24	367302	0.06	0.23	357942
Dependency Ratio	106.04	87.99	364440	133.37	98.15	353176
Primary Education (Head)	0.77	0.42	367292	0.57	0.50	357942
Unemployment Rate (Constituency)	10.60	4.20	367302	4.12	2.79	357942
Agriculture (Constituency)	48.23	27.62	367302	81.23	21.21	357942
Manufacturing (Constituency)	15.92	9.65	367302	5.16	5.24	357942
Services (Constituency)	35.66	18.56	367302	13.53	16.20	357942
Other Unknown Industry (Constituency)	0.19	0.34	367302	0.08	0.15	357942
Skilled 19–64 yrs (Constituency)	14.96	6.69	367302	6.42	5.37	357942
Unskilled 19–64 yrs (Constituency)	82.85	7.18	367302	90.86	6.59	357942
Unknown skill 19–64 yrs (Constituency)	2.19	1.40	367302	2.72	2.34	357942

**Table A2:** Effect of Copper Boom on Adult Marriage

	(1) Pooled	(2) Pooled	(3) Pooled	(4) Male	(5) Female
Treatment	0.019 (0.025)	0.028 (0.025)	−0.019 (0.026)	−0.009 (0.020)	−0.016 (0.031)
Primary Education Head	0.100^***^ (0.007)	0.100^***^ (0.007)	0.058^***^ (0.011)	0.080^***^ (0.012)	0.030^**^ (0.011)
Male	−0.005^*^ (0.003)	0.011^*^ (0.006)	−0.005^*^ (0.003)		
Male X Treatment		−0.018^**^ (0.007)			
Primary Educated Head X Treatment			0.056^***^ (0.012)	0.053^***^ (0.013)	0.048^***^ (0.013)

Constituency FE	Yes	Yes	Yes	Yes	Yes
Observations	75290	75290	75290	37693	37597
Mean Dep. Variable	0.268	0.268	0.268	0.259	0.276
SD Dep. Variable	0.443	0.443	0.443	0.438	0.447

The treatment group is 30km and the control group is 30–100km of a copper mine between 1990 and 2000. The sample is for 2010. The dependent variable is the indicator for marriage after 2003, zero otherwise. There are individual-level, household-level and constituency-level labor market controls included in the above regressions. The individual-level variables include age and gender of the child. Household-level variables include the dependency ratio and head’s education level. Constituency-level labor market variables include unemployment rate, share of employment in different sectors and skill level of the workers.

* p < 0.1

** p < 0.05

*** p < 0.01.
